# Protocol for evaluation of the continuum of primary care in the case of a miscarriage in the emergency room: a mixed-method study

**DOI:** 10.1186/s12884-017-1309-5

**Published:** 2017-04-20

**Authors:** Francine de Montigny, Chantal Verdon, Diane Dubeau, Annie Devault, Martin St-André, Éric Tchouaket Nguemeleu, Carl Lacharité

**Affiliations:** 1 Universite du Quebec en Outaouais, Gatineau, QC Canada; 2 Université du Quebec en Outaouais, St-Jérôme, QC Canada; 3 St-Justine Hospital, Montreal, QC Canada; 4 Université du Quebec a Trois Rivieres, Trois Rivieres, QC Canada

**Keywords:** Emergency, Healthcare services, Institutional inscription, Mental health, Miscarriage, Perinatal grief

## Abstract

**Background:**

In Quebec (Canada), nearly 20,000 pregnancies end in miscarriage, and the majority of the miscarriages are dealt with in an emergency unit. Although there are studies documenting the effects of this type of grief on mental health, men’s experiences are much less discussed than those of women. Similarly, no study has evaluated best practices in terms of service continuity, from emergency care to community resources. The aim of this study is to better understand the relationships that exist between the organization of emergency room and primary care health services for women presenting with miscarriage, on the one hand, and the positions and experiences of women and men within these services, on the other.

**Methods:**

The general objective of this mixed-method study can be broken down into three methodological sections. *Focus 1. Institutional discourses and practices.* This section is structured as a multiple case study of the mandates of five participant institutions. The study will involve (a) a documentary analysis; (b) a quantitative survey (N: 200) and (c) group interviews (N: 75) with caregivers and emergency unit managers. *Focus 2. Women’s and men’s experiences of miscarriages and the institutional response.* This section includes (a) a survey (N: 232) and (b) individual interviews (N: 80) designed to identify best practices in emergency involving women and their partners in each area. *Focus 3*. This section will integrate the information furnished by the first two sections in order to create an *ethnographic overview* of the situation.

**Discussion:**

This innovative project will provide answers to critical questions on how to improve the effectiveness and quality of interdisciplinary and multisectoral interventions to promote the mental health and psychosocial well-being of couples having experienced a miscarriage. It will have a material effect on the organization of emergency services and of the primary care pathway for women experiencing a miscarriage and for their partners.

**Trial registration:**

Not applicable. This study involves a retrospective view of usual health care interventions. This study is not a clinical trial that prospectively assigns human participants or groups of humans to one or more health-related interventions to evaluate the effects on health outcomes*.*

## Background

It is estimated that 25% of pregnancies terminate otherwise than in a live birth [1]. In Quebec (Canada), this amounts to around 20,000 pregnancies each year. However, the data available under-represent the real number of losses occurring before the twentieth week of pregnancy, as these rarely require that women be hospitalized. These early losses, known as miscarriages, are not currently reflected in Canadian statistics on pregnancy. Despite the significant prevalence of loss during pregnancy, the bereavement it causes has always garnered less attention, in scientific and professional communities, than that resulting from other forms of death (for example, suicide). Yet, the issue of bereavement following an early perinatal loss is a major one in our society.

An early loss leads to increased risk among women of developing a major depressive episode (2.5 times greater risk) [2] or a minor depressive episode (5 times greater risk) [3, 4] that can last several years. The prevalence of somatization, various anxiety disorders, or obsessive compulsive disorders is also higher in the year after having experienced a miscarriage [5]. As for men, although their experience is considerably less documented than that of women, it is known that the sadness they feel is just as intense [6]. Their depressive symptoms are highest, however, at 30 months after the loss, much later than for women [7]. There is also a decline in conjugal satisfaction after a perinatal death, a deterioration in couples’ relationships that can even result in separation or divorce [8, 9]. Relationships with children born following a miscarriage can also be disrupted. Examples of this are the high rate of disorganization observed in mother–child attachment among children born after a perinatal death [10] and studies on “replacement children” [11, 12] in which parents were seen to have difficulty investing in children born after a perinatal death, which can impair children’s identity development or even increase the risks of different psychopathologies or relationship problems [11].

Even though the experience and repercussions of perinatal bereavement following an early pregnancy loss have been studied, couples’ experience of professional support – or of the absence of support – is seldom addressed. The few studies on the experience of practitioners who provide counselling for pregnancy loss show that it is demanding for professionals to support couples in bereavement [13–15]. Authors point to health professionals’ lack of time and training [16, 17], deficiencies that affect the support and information given to couples and contribute to their isolation and distress, in addition to prolonging their bereavement [16].

In Quebec, there are no specific protocols or directives for providing care to women who present symptoms of complications in early pregnancy (<20 weeks) [18]. In the emergency room, these women are generally considered “stable”, which means they can expect a significant wait before their first medical assessment. On being discharged, they are referred to their family physician and are advised to return to emergency if bleeding increases. The number of readmissions to emergency is not known exactly, as care for miscarriage in the emergency room has never been evaluated. Finally, these women are not systematically referred to community primary care services following their miscarriage.

### Study purpose

The aim of this study is to better understand the relationships that exist between the organization of emergency room and primary care health services for women presenting with miscarriage, on the one hand, and the positions and experiences of women and men within these services, on the other. More specifically, this study will enable us to:

- Focus 1: Critically describe the component elements of institutional practices and discourse to see how couples and their services pathway through the miscarriage experience are “constructed” within professional mandates.
- Focus 2: Describe the experiences of women undergoing a miscarriage in the emergency room, and those of their partners, with respect to treatment and the care continuum.
- Focus 3: Construct an explanation (or a theory) of institutional action in cases of miscarriage and of how men and women become enrolled in primary care health and social services.

## Methods/Design

### Study design

Our study will require both objective (quantitative data) and subjective (qualitative data) investigation into the experiences of women, men, and practitioners, which necessarily means using a mixed-method design. The methodological approach employed relies on embedded multiple-case study design [19]. This approach is especially useful to gain a deeper understanding of a phenomenon such as miscarriage situations, by documenting in greater detail the processes unfolding within specific social contexts.

Two hypotheses will be explored. Hypothesis 1: The quality of the conjugal relationship after a miscarriage is associated with a positive mental health trajectory, the relationship being mediated by two variables: the quality of services and the quality of relationships with health professionals. Thus, the higher the quality of services and of relationships with health professionals reported by couples, the healthier their mental health trajectory will be, even in adverse personal or contextual conditions. Hypothesis 2: The factors associated with the mental health trajectories of men and women differ according to: a) personal characteristics, e.g. attitude with respect to services, age, education, previous losses, physical and mental health, and the presence of other children; b) contextual characteristics, e.g. perception of social support (formal and informal), income level (low, versus average or high), and immigration status.

### Guiding framework

The questions deriving from the study’s objectives and hypotheses can be examined through various conceptual filters.

#### Focus 1

**Fig. 1:**
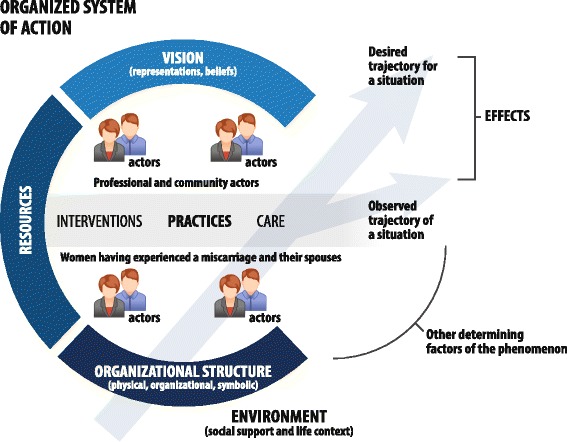
A model of primary care services as an organized system of action

We begin with Contandriopoulos and colleagues’ [20] conception of primary care services as an organized system of action (Fig. 1). Thus, all primary care services (notably emergency services) in a given context (a region), at a given time, have structures (physical, organizational, symbolic) that influence the activities of actors (health professionals, couples) to maintain a positive health trajectory for couples (mental and physical health).

#### Focus 2

**Fig. 2:**
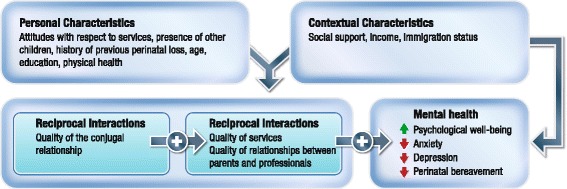
Conceptual framework of men’s and women’s individual experience of the services and health trajectory

The bioecological model of human development [21] further grounds our examination of the development of mental health trajectories, by taking into consideration interactions between individuals and persons in their near environment (spouse, health professionals) and those at a distance (policies, healthcare services restructuring) as engines for this development (Fig. 2). Personal characteristics (age, education, presence of other children, history of previous losses, attitudes regarding mental services, physical and mental health) and contextual characteristics (income, social support, immigration) are the remaining elements likely to influence the mental health trajectory.

#### Focus 3

Finally, an especially useful conceptual and methodological framework for studying the relationships between the organization of healthcare services and the experience of couples (actors) who rely on them is institutional ethnography (IE) [22–26]. IE examines the way in which persons come to belong to a given institution. It focuses on social relationships established between persons within the institutional space as well on the forms of regulation that shape these relationships. IE is an approach that provides insight into the connections between local practices implemented by people, on one hand, and translocal policies, regulations, and rules, on the other. The expression “institutional inscription”, coined by Smith [26], is used here to refer to the process through which people position themselves within the relationships of regulation, production, and affectivity shaped by the institutional context in which they are participating [27]. IE also takes into account gender relations in the participation of men/fathers and women/mothers within both the family and the health services context [24, 28–31]. It is critical to emphasize here that this approach does not simply aim to describe reality in a positivist manner, but also involves a critique of the real. In this respect, it provides a means to interpret the data collected based on what could become real if appropriate and timely actions were taken.

### Participant recruitment and data collection

The study will take place in five regions, representing 80.9% of the population of Quebec and 81.2% of births [32]. Five (*N* = 5) institutional participants (health and social services centres) have been selected for the diversity of their population (urban, semi-urban, and rural), the number of miscarriages recorded annually, and the disparity and diversity of services in the regions.

The recruitment and data collection procedures are described in Tables 1 and 2. To participate in the study, practitioners and managers must work in emergency services at one of the five participating institutions. As for parents, they must have visited the emergency room of one of the five participating institutions within the past 12 months and have received one of the following diagnoses: a) a threatened miscarriage terminating in a miscarriage; b) an inevitable, incomplete, or complete miscarriage; or c) an ectopic pregnancy. Moreover, the pregnancy needs to be 20 weeks or less, the father needs to be the biological parent of the child, and both partners must be over 18 years old and able to speak, understand, and read French.

**Table 1:** Recruitment and data collection process – Focus 1

Sources/participants	Recruitment method	Instruments
A) Documentary data		
1. Framework programs and practice statements 2. Protocols and data collection guidelines 3. Medical charts (N: 200)	1. All documents 2. All documents 3. Charts randomly selected by archivist (N: 40/establishment)	3. Chart analysis grid: a. sociodemographic data b. gestational status c. triage score d. resources mobilized (e.g. blood tests, ultrasounds, medication, etc.) e. organizational structures (e.g. waiting time, length of stay, number of admissions and readmissions, etc.)
B) Online survey		
N: 200 emergency services practitioners	Advertising in the workplace	a. Sociodemographic data b. Families’ Importance in Nursing Care scale [33] c. Self-efficacy at Work Scale [34] d. Attitudes to Perinatal Bereavement Care Scale [35] e. Organizational structure for providing care to couples experiencing a miscarriage (grid based on the documentary analysis)
C) Group interview		
N: 75 emergency service practitioners and managers	Advertising in the workplace	Interview guide based on the narrative approach [36, 37]

**Table 2:** Recruitment and data collection process – Focus 2

Sources/participants	Recruitment method	Instruments
A) Online surveys		
N: 232 parents^a^	Advertising on social media, support groups, medical clinics and key emergency room informants	a. Sociodemographic data b. Institutional support evaluation (unpublished document) c. Index of Emotional Well-being [38, 39] d. Dyadic Adjustment Scale [40] e. Edinburgh Postnatal Depression Scale [41] f. State Anxiety Inventory [42] g. Perinatal Grief Scale – Short Version [43]
B) Individual interviews		
N: 80 parents Group 1: 20 couples, first perinatal loss Group 2: 20 couples, previous perinatal loss	Sampled from those who indicated interest in the online survey	Interview guide based on critical incident technique [44]

^a^An a priori calculation of statistical power shows that a minimum sample of 232 parents is necessary to achieve the following criteria: effect size (multivariate) = 0.15; number of predictive variables = 11; significance threshold = 0.05; required power = 0.99

### Data analysis

#### Qualitative data (group and individual interviews)

Each individual and group interview will be transcribed and then coded and analyzed according to emergent themes. To ensure the quality of the coding and analysis, 20% of the corpus of transcripts will be cross-coded and analyzed by two independent researchers to obtain interrater agreement.

#### Quantitative data (surveys)

Descriptive and correlational analyses will highlight the most salient elements of practitioners’ and couples’ experiences. Logistic regression will help document the specific effects of the independent variables being studied. A theoretical model of the organization of actions (practitioners) and of care and services pathways (couples) will be verified using a structural equation modelling technique. We will use these analyses to explore the nature of the relationships between the variables being studied.

#### Ethnographic analysis

By integrating the data collected, we should be able to build an ethnographic overview of the treatment of women and their partners experiencing a miscarriage in the emergency room. The conceptual framework described above provides several elements that will shape the development of a framework for analysis of the data to be collected during this study. First, documentary information will be analyzed to bring to light the explicit and implicit organizing principles that “govern” the field of practice at each site. The results of this analysis will translate into a position paper for each site. These relatively succinct documents (5 to 8 pages) will be submitted to key actors (practitioners and managers) for validation and, as needed, will be modified following an iterative process (ecological validation). These documents will serve as local reference frameworks for interpreting all of the other data collected.

Second, the empirical data (survey results, group interviews, interviews with women and their partners) from each site will be analyzed concurrently to produce *informational summaries* (conceptual maps based on the interviews, documents, and healthcare services data).

Third, these summaries will be merged to produce, for each site, a *diagram of the institutional inscription of women and their partners experiencing a miscarriage in the emergency room*. These local diagrams will also be validated by key actors. Finally, the last level of analysis will focus on integrating all of the institutional inscription diagrams into a comprehensive overview incorporating convergences and distinctions.

### Ethical considerations

This research protocol has been approved by the research ethics committees of the Université du Quebec en Outaouais (Reference number 1799) and of the participating institutions, as applicable (Reference numbers 2013–116; 2013-286-E; CER-1314-031; B14-02-19191).

## Discussion

The originality of the present study lies in adopting a conceptual framework that allows for a complete analysis of institutional perceptions of professional practices with respect to women and their partners experiencing a miscarriage in the emergency room, as well as of men’s and women’s statements regarding their experience of their care and services trajectory. It also comes from the attention given to the experience of men in a context where scientific knowledge and currently implemented interventions mostly focus on women. Moreover, our analysis of the care provided to women and their spouses during a miscarriage in Quebec will be significantly enriched by triangulating the different views on the service trajectories of men and women (views of men, women, and practitioners) and the methodologies (quantitative and qualitative). Finally, our analysis of the care provided for women experiencing a miscarriage and of the physical and mental health trajectory of the couple will take into account the accessibility, efficiency, productivity, continuity, responsiveness, and quality of primary care.

By establishing close ties between theory, research, and practice, this large-scale multicentre project should lead to greater social and public health relevance for the research conducted and, consequently, to a greater probability that the findings from our work will have an impact not only within the scientific community, but also in the professional and even civil communities. Moreover, our methodological choices foster greater sensitivity to the complexity of the contexts, or ‘landscapes’, of practice settings that come into daily contact with women, men, families, and local communities. By participating in this project, emergency room practitioners and managers will have the opportunity to get some perspective on their own organizational methods and the role they play in the institutional response with respect to women and their partners, which could have a structuring effect on the organization of practices and of services provided to women experiencing a miscarriage and their partners.

Convenience sampling is a study limitation, however. Although it improves the study’s feasibility, it is possible that this approach will favour persons who may have suffered more or people who are more open to sharing their experience. The large sample size should mitigate this limitation.

### Expected impact

This study will generate a great quantity of data concerning the nature of the relationships that exist between couples who have experienced a miscarriage and the health and social services professionals who work with them across a wide range of emergency services in Quebec. To our knowledge, few studies have produced this type of data and no research on this topic has been conducted within Quebec or Canada. The body of knowledge resulting from this project will guide construction of intervention models for primary healthcare and social services in Quebec, including emergency services. Orientations, clinical principles, and guidelines focusing specifically on promoting a positive health trajectory in cases of a miscarriage, with particular attention to the experience of men, will be proposed, in response to the real needs of couples. Finally, this project is expected to lead to the production of a reference book for this field, aimed at professionals and students, regarding professional practices with respect to bereaved families.

This innovative project will provide answers to critical questions on how to improve the effectiveness and quality of interdisciplinary and multisectoral interventions to promote the mental health and psychosocial well-being of couples having experienced a miscarriage. It will have a material effect on the organization of emergency services and of the primary care pathway for women experiencing a miscarriage and for their partners.

## Abbreviations

IE: Institutional ethnography
